# POLICY SERIES: STRATEGIES TO FRAME OUR PUBLIC POLICY WORK

**DOI:** 10.1093/geroni/igad104.0285

**Published:** 2023-12-21

**Authors:** Patricia D’Antonio, Julie Schoen, Hannah Albers

## Abstract

Our words and the way we harness the power of them when advocating for age inclusive policies, like the Older Americans Act (OAA), matters. Since 1965 the OAA has supported our nation with funds to strengthen community social services. It currently authorizes a wide array of service programs through a national network of 56 state agencies on aging, 618 area agencies on aging, nearly 20,000 service providers, 281 Tribal organizations, and 1 Native Hawaiian organization representing 400 Tribes. In 2024, the OAA will be considered for reauthorization. We all have a stake in ensuring it is passed so we all have the infrastructure we require as we age. Join The National Center to Reframe Aging and the National Center on Elder Abuse, the leading organizations for effective framing, in this session to discuss the OAA as an example of how we can apply communication strategies in supporting reauthorization, review framing strategies to advance a more equitable story of aging in America and learn how to apply framing tools to your advocacy communications.

